# Supplementary material for: One-Step Synthesized Iron-Carbon Core-Shell Nanoparticles to Activate Persulfate for Effective Degradation of Tetrabromobisphenol A: Performance and Activation Mechanism
Source: Nanomaterials (Basel). 2022 Dec 18;12(24):4483. doi: 10.3390/nano12244483 (PMC9787185; doi:10.3390/nano12244483)
Supplement: Supplementary file 1 [file nanomaterials-12-04483-s001.zip › nanomaterials-2067105-supplementary.pdf]

nanomaterials

One-Step Synthesized Iron-Carbon Core-Shell Nanoparticles to Activate Persulfate for Effective Degradation of Tetrabromobisphenol A: Performance and Activation Mechanism

Yunjiang Yu [1], Chang Liu [1,2], Chenyu Yang [1], Yang Yu [3], Lun Lu [1], Ruixue Ma [1], and Liangzhong Li [1,*]

1 State Environmental Protection Key Laboratory of Environmental Pollution Health Risk Assessment, Center for Environmental Health Research, South China Institute of Environmental Sciences, Ministry of Ecology and Environment, Guangzhou 510655, China
2 Inner Mongolia Autonomous Region Key Laboratory of Water Pollution Control, School of Ecology and Environment, Inner Mongolia University, Hohhot 010021, China
3 Guangdong Key Laboratory of Environmental Pollution and Health, School of Environment Jinan University, Guangzhou 511443, China
* Correspondence: liliangzhong@scies.org

Table S1. The summaries of reported organic pollutants degradation conditions and performances by iron-based activated PS degradation system.

Table S2. The basic information of the actual contaminated water.

Figure S1 SEM and TEM image of Fe@MC.

Figure S2 Hysteresis curves of Fe@MC nanocore-shell catalysts.

Figure S3 TGA of Fe@MC .

Figure S4 Full XPS spectrum Fe@MC.

Figure S5 The relative content of C, O, Fe analyzed by SEM-EDX.

Figure S6 N_2 sorption isotherms and pore size distributions of Fe@MC .

Figure S7 Comparison of the effect of Fe@MC/PS system and Fe@MC on removing TBBPA.

Figure S8 Recyclability and regeneration of Fe@MC core-shell nanoparticles on the degradation of TBBPA.

Figure S9 The removal of TBBPA by Fe@MC/PS in actual contaminated water(a) and TOC removal removal efficiency with different PS concentration (b).

Figure S10 Electron density Laplacian analysis (a) and projection map of electron localization function ELF (b) of TBBPA.

Figure S11 Corresponding mass spectra and proposed structure of degradation products of TBBPA.

Table S1. The summaries of reported organic pollutants degradation conditions and performances by iron-based activators.

Catalyst	（g/L）	pollutant	(mg/L)	PS/PDS	pH	Reaction Time (min)	Removal efficiency (%)	Reference
Fe@MC	0.2	TBBPA	10	2.0	7.0	10	94.9	This work
MCFe	0.5	2,4,6-TCP	100	2.0	3	180	94.8	[1]
Fe@CN	0.1	BPA	20	0.5	6.8	60	96.0	[2]
Fe/Co@mHS	1	TBBPA	10	20.0	5	6	97.13	[3]
AC@Fe_3O_4	0.4	TC	10	40	3	180	99.8	[4]
Fe0@Fe_3O_4	0.5	DPB	5	1.8	7	180	94.7	[5]

Table S2. The basic information of the actual contaminated water.

pH	Electrical conductivity ($ms \cdot cm^{-1}$)	COD ($mg \cdot L^{-1}$)	TOC ($mg \cdot L^{-1}$)	Turbidity (NTU)
8.22	1.10	202.0	160.1	87

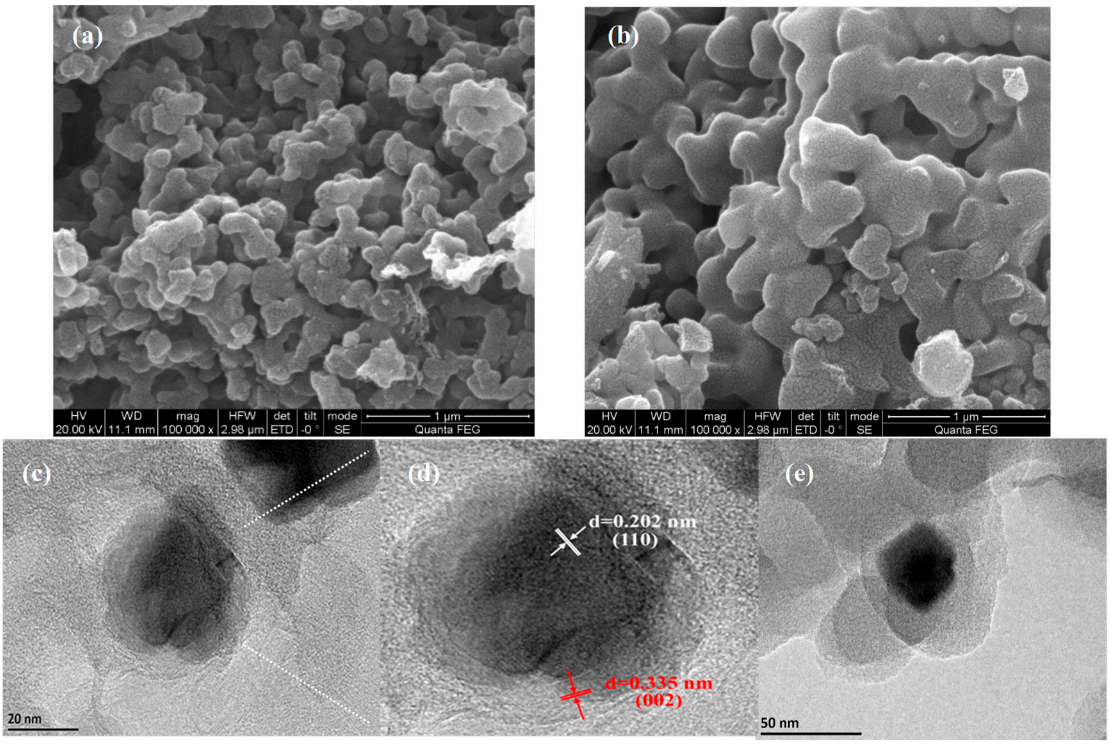

Figure S1. SEM (**a** 800 °C, **b** 1200 °C) and TEM image of (**b,c,d**) Fe@MC.

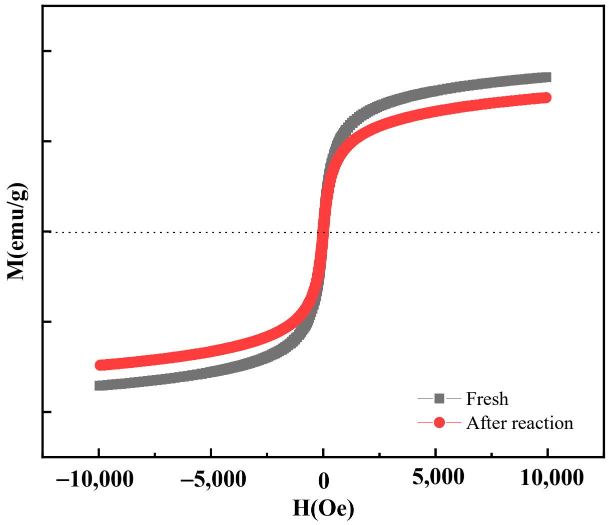

Figure S2. Hysteresis curve image of Fe@MC

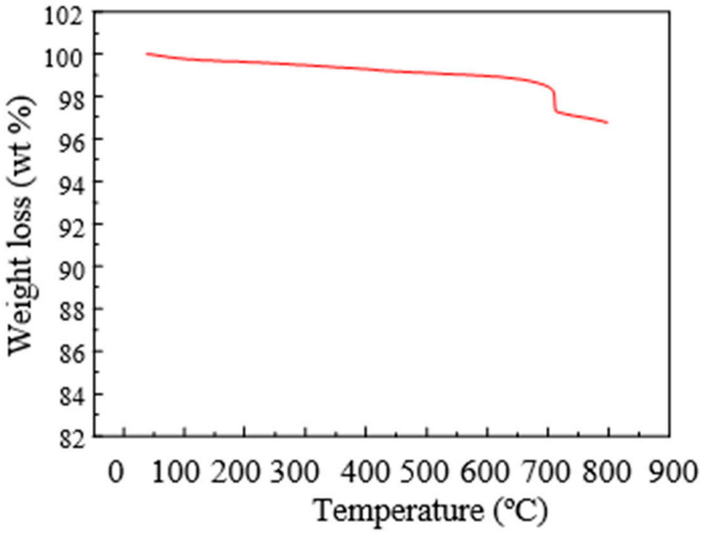

Figure S3. TGA of Fe@MC.

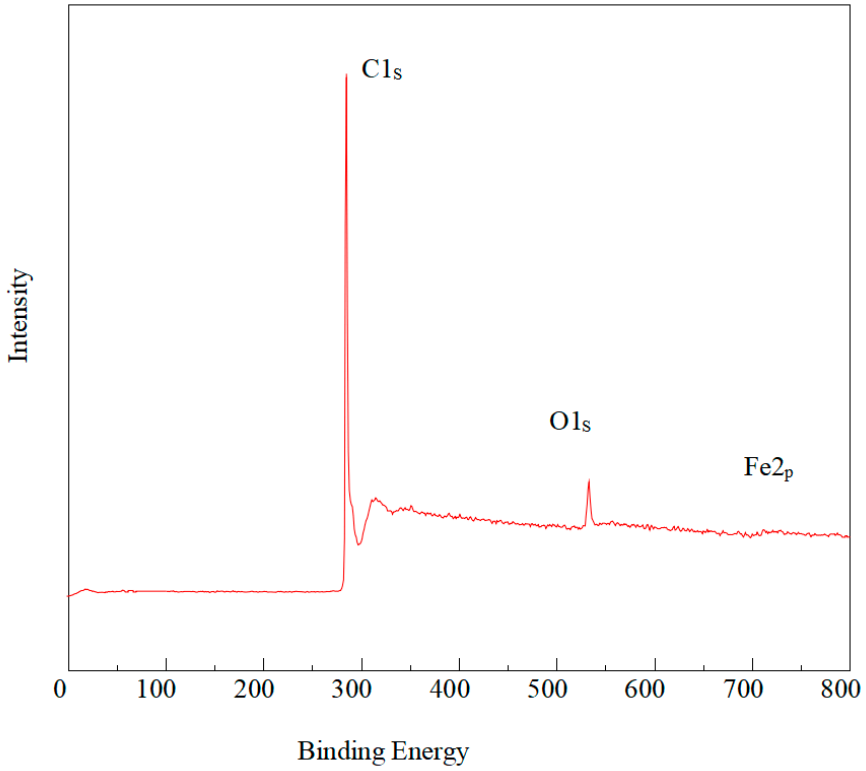

Figure S4. Full XPS spectrum Fe@MC .

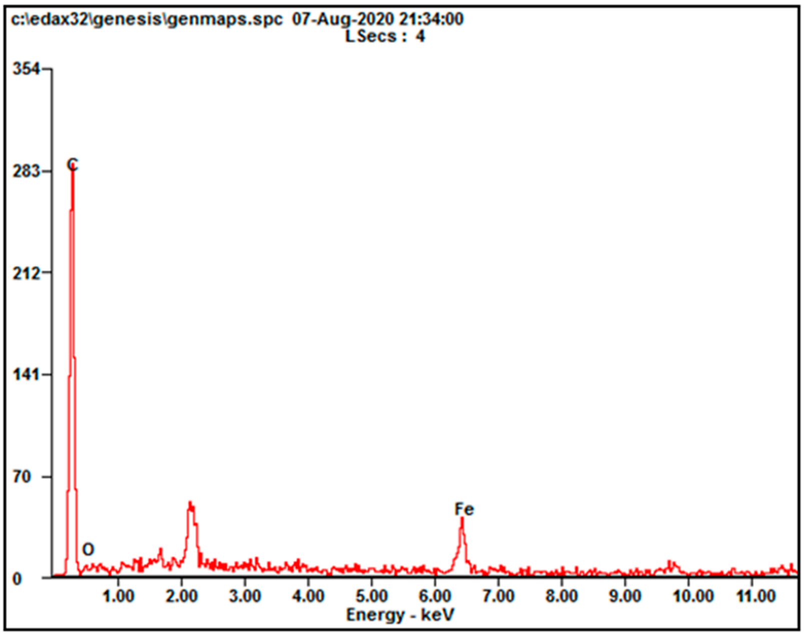

	Wt%	At%
CK	88.49	95.77
OK	02.67	02.17
FeK	08.84	02.06
Matrix	Correction	ZAF

Figure S5. The relative content of C, O, Fe analyzed by SEM-EDX.

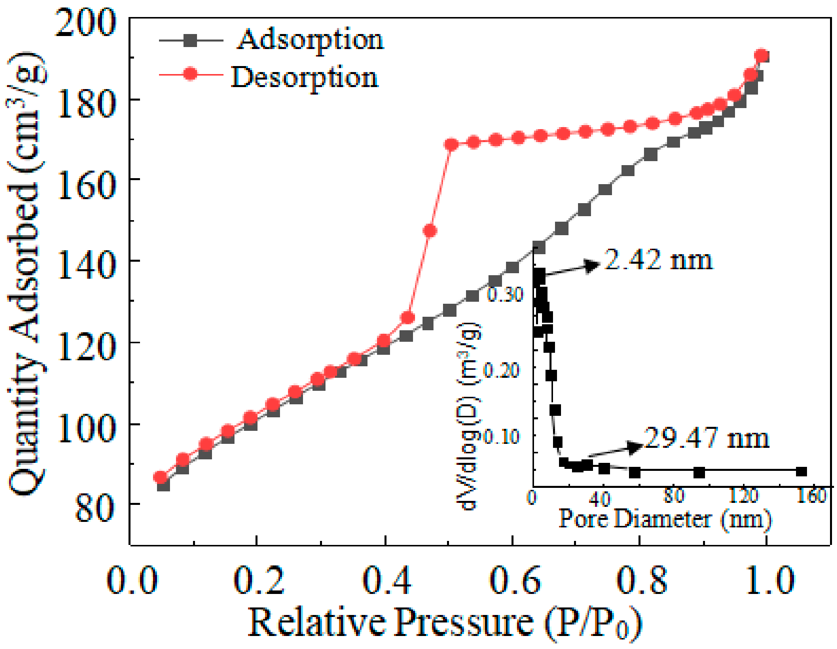

Figure S6. N_2 adsorption-desorption isotherms and pore size distributions of Fe@MC.

Figure S7.

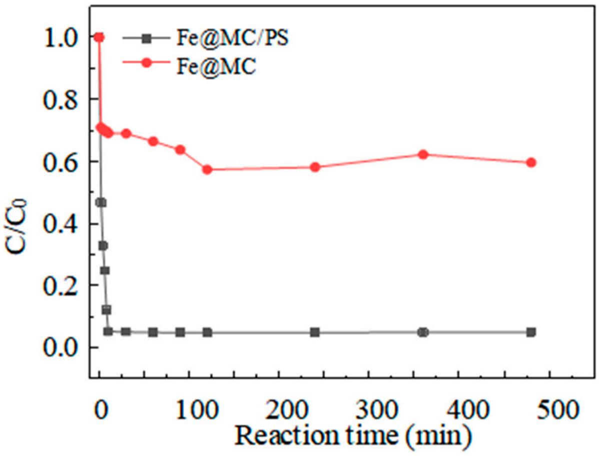

.

Figure S7. Effect of PS Addition on Removal of TBBPA

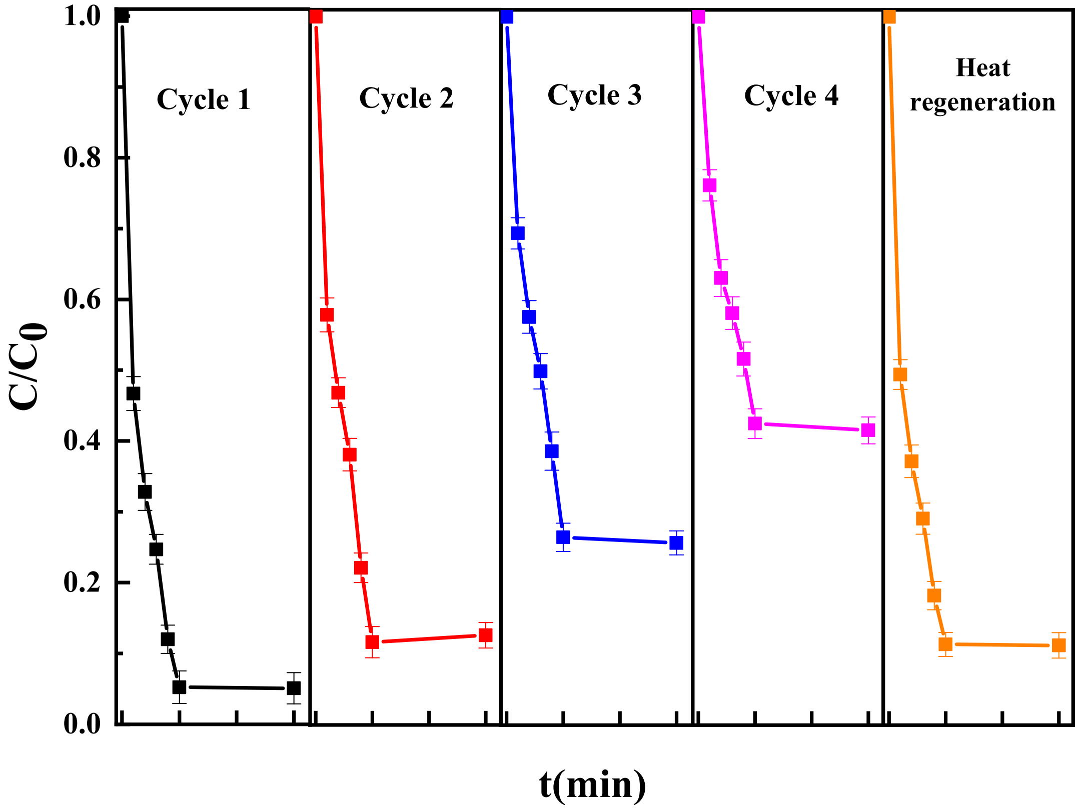

Figure S8. Recyclability and regeneration of Fe@MC

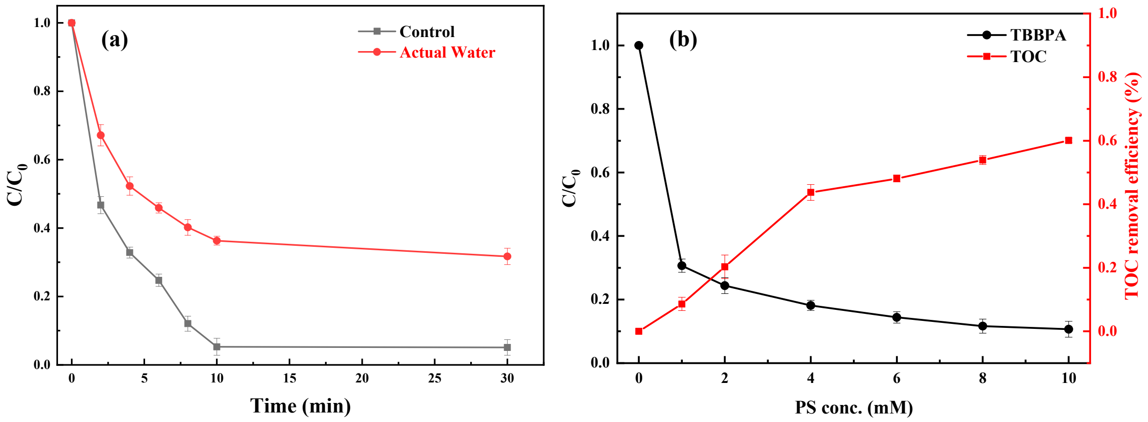

Figure S9. The removal of TBBPA by Fe@MC/PS in actual contaminated water(**a**) and TOC removal removal efficiency with different PS concentration (b).

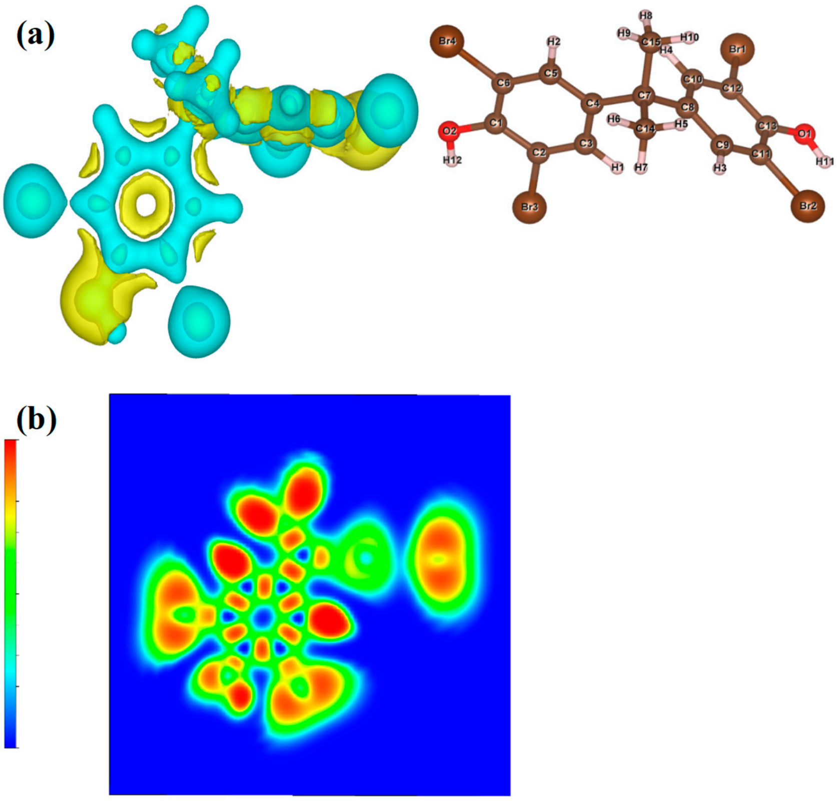

Figure S10. Electron density Laplacian analysis (**a**) and projection map of electron localization function ELF (**b**) of TBBPA.

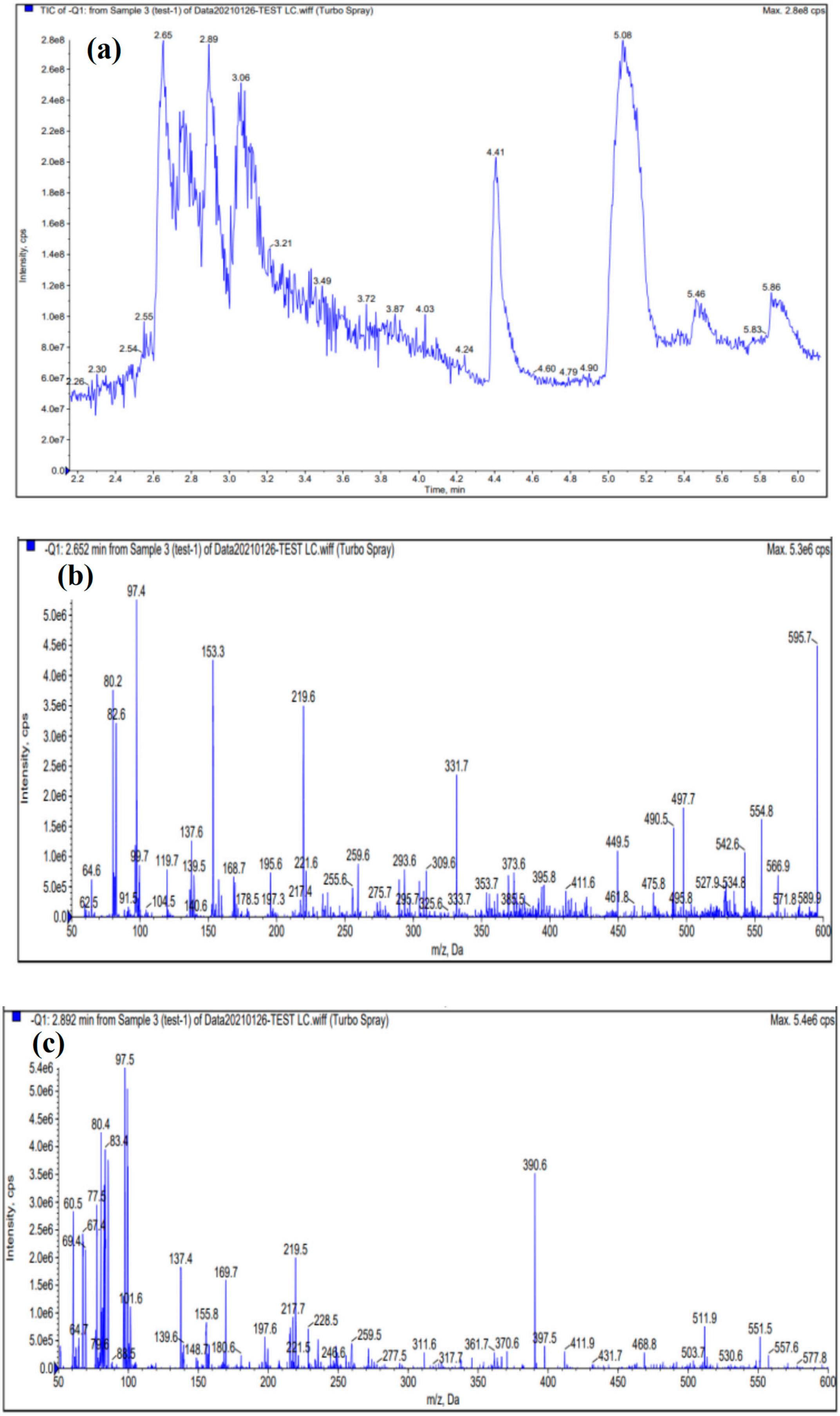
(a)
TIC of -Q1: from Sample 3 (test-1) of Data20210126-TEST LC.wiff (Turbo Spray)
Max. 2.8e8 cps
Intensity, cps
Time, min
(b)
-Q1: 2.652 min from Sample 3 (test-1) of Data20210126-TEST LC.wiff (Turbo Spray)
Max. 5.3e6 cps
Intensity, cps
m/z, Da
(c)
-Q1: 2.892 min from Sample 3 (test-1) of Data20210126-TEST LC.wiff (Turbo Spray)
Max. 5.4e6 cps
Intensity, cps
m/z, Da

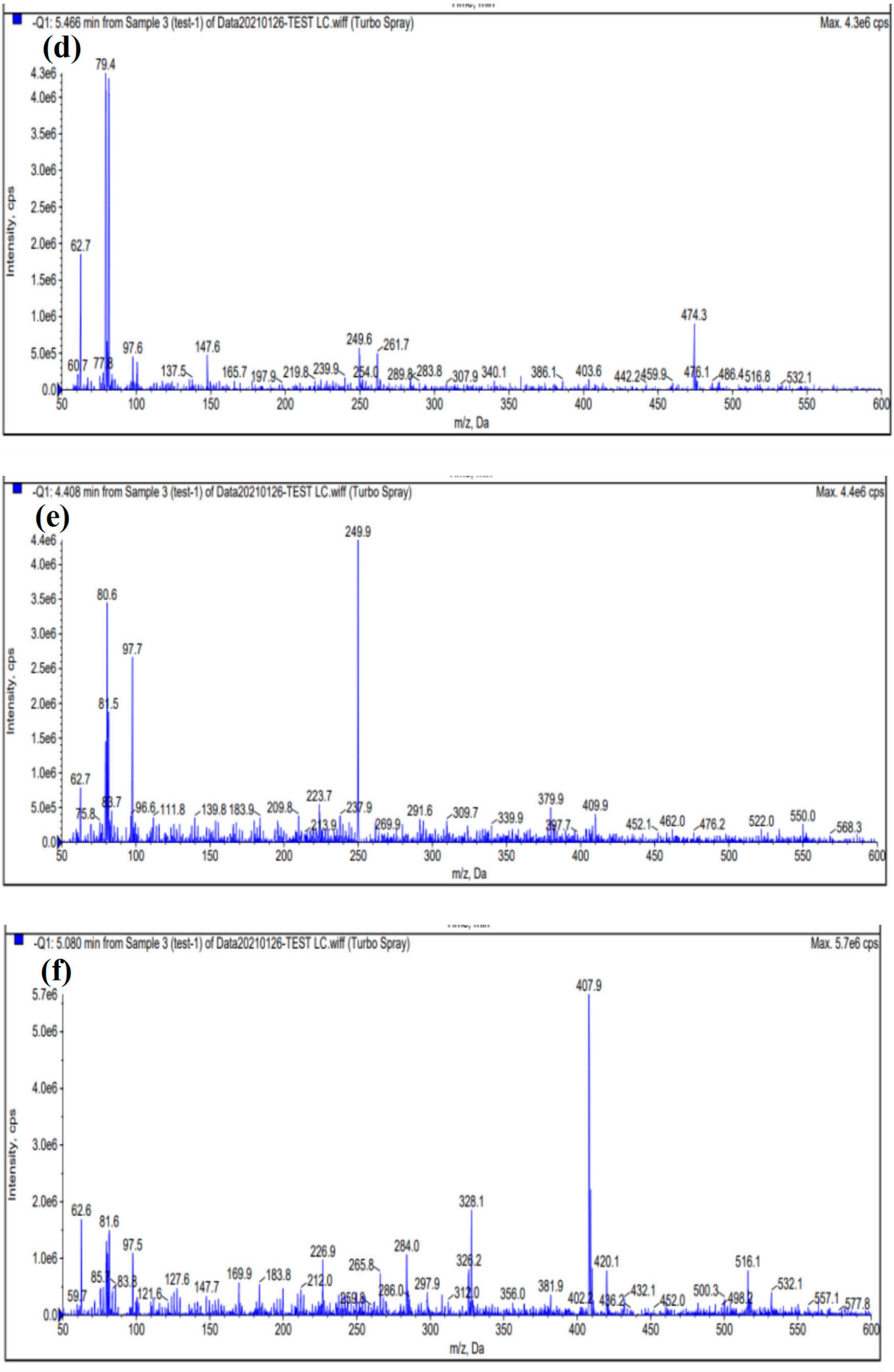

Figure S11. Corresponding mass spectra and structure of degradation products of TBBPA(The total mass spectra of degradation products (a) and each degradation product mass spectra (b,c,d,e,f)).

1. Wu, Y.W.; Chen, X.T.; Han, Y.; Yue, D.T.; Cao, X.D.; Zhao, Y.X.; Qian, X.F. Highly Efficient Utilization of Nano-Fe(0) Embedded in Mesoporous Carbon for Activation of Peroxydisulfate. *Environ. Sci. Technol.* **2019**, *53*, 9081–9090.
2. Liu, L.; Xu, X.; Li, Y.w.; Su, R.D.; Li, Q.; Zhou, W.Z.; Gao, B.Y.; Yue,Q. Y. One-step synthesis of "nuclear-shell" structure iron-carbon nanocomposite as a persulfate activator for bisphenol A degradation. *Chem. Eng. J.* **2020**, *382*, 122780.
3. Xiang, M.H.; Huang, M.F.; Li, H.; Wang, W.B.; Huang, Y.; Lu, Z.; Wang, C.; Si, R.F.; Cao, W. Nanoscale zero-valent iron/cobalt@mesoporous hydrated silica core–shell particles as a highly active heterogeneous Fenton catalyst for the degradation of tetrabromobisphenol A. *Chem. Eng. J.* **2021**, *417*, 129208.
4. Li, H.X.; Wan, J.Q.; Ma, Y.W.; Yan, W. Synthesis of novel core–shell Fe@Fe_3O_4 as heterogeneous activator of persulfate for oxidation of dibutyl phthalate under neutral conditions. *Chem. Eng. J.* **2016**, *301*, 315-324.
5. Jonidi, J.A.; Kakavandi, B.; Jaafarzadeh, N.; Rezaei, K,R.; Ahmadi, M.; Akbar, B,A. Fenton-like catalytic oxidation of tetracycline by AC@Fe_3O_4 as a heterogeneous persulfate activator: Adsorption and degradation studies. *Ind. Eng. Chem.* **2017**, *45*, 323-333.
